# Challenges of scoliosis management in bilateral upper limb amelia: a case report of selective thoracic fusion

**DOI:** 10.1093/jscr/rjag582

**Published:** 2026-07-13

**Authors:** Priyanshu Saha, Husam Aldin Jassemi Zergani, Matthew Ariya Arden, Febisayo Sidiku, Jason Bernard, Timothy Bishop, Darren F Lui

**Affiliations:** Department of Complex Spine Surgery, St George’s University Hospitals NHS Foundation Trust, London SW17 0QT, United Kingdom; Department of Complex Spine Surgery, St George’s University Hospitals NHS Foundation Trust, London SW17 0QT, United Kingdom; Department of Complex Spine Surgery, St George’s University Hospitals NHS Foundation Trust, London SW17 0QT, United Kingdom; Department of Complex Spine Surgery, St George’s University Hospitals NHS Foundation Trust, London SW17 0QT, United Kingdom; Department of Complex Spine Surgery, St George’s University Hospitals NHS Foundation Trust, London SW17 0QT, United Kingdom; Department of Complex Spine Surgery, St George’s University Hospitals NHS Foundation Trust, London SW17 0QT, United Kingdom; Department of Complex Spine Surgery, St George’s University Hospitals NHS Foundation Trust, London SW17 0QT, United Kingdom

**Keywords:** amelia, congenital limb absence, scoliosis, selective thoracic fusion, functional preservation

## Abstract

Amelia is a rare congenital malformation characterized by the complete absence of one or more limbs. We report the case of a 17-year-old boy with bilateral upper limb amelia and progressive thoracic scoliosis. He was fully independent in daily living, relying on his lower limbs for all activities. Radiographs demonstrated an 86° thoracic curve (T6–T12), skeletal maturity, and limited flexibility. Theoretical fusion to L3 or L4 risked major functional decline. He underwent selective thoracic fusion (T3–L1), sparing four lumbar discs. At 2-year follow-up, Cobb angle improved to 34°, SRS-22 score rose from 2.8 to 3.55, and independence was preserved. This case highlights the need to balance deformity correction with functional preservation in scoliosis management for patients with amelia.

## Introduction

**Figure 1:**
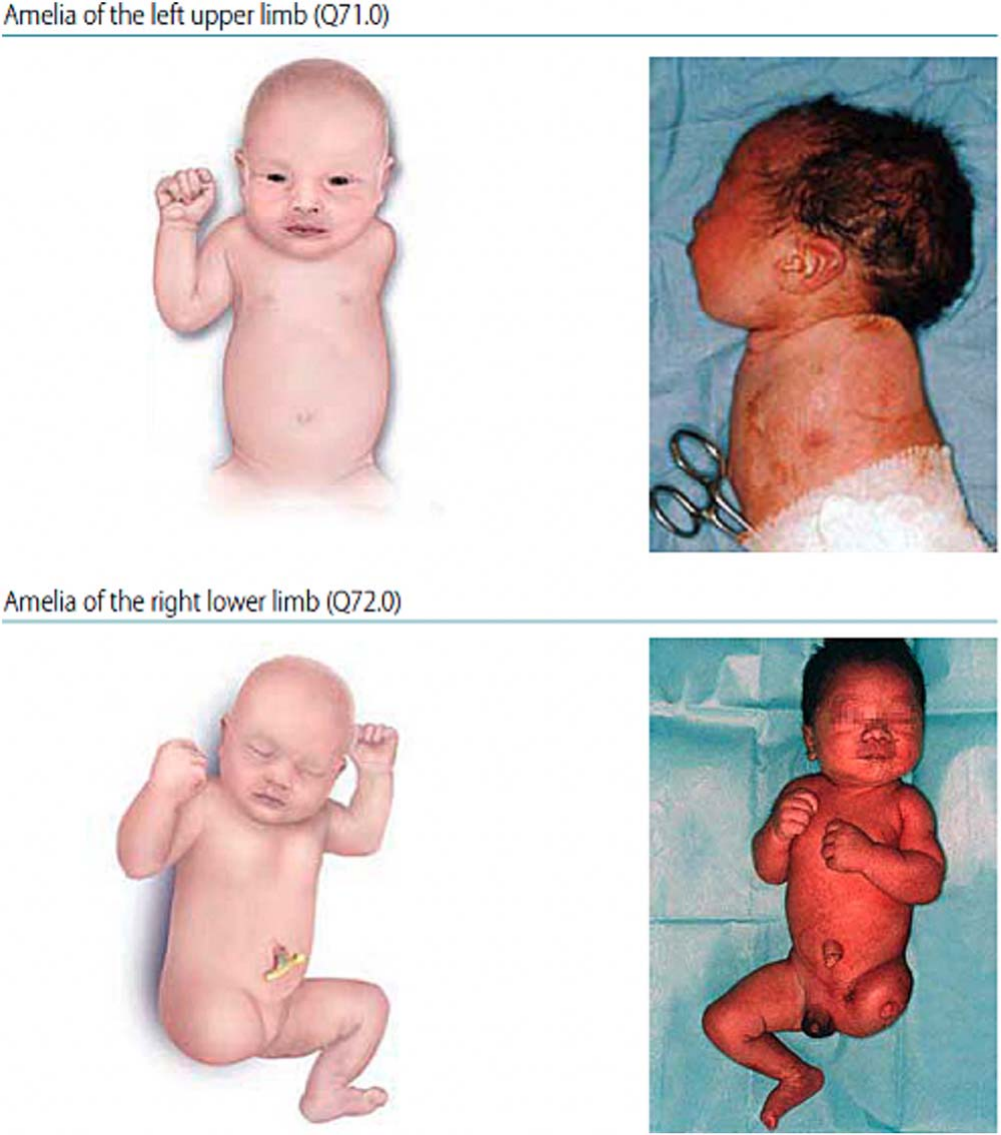
Clinical presentation of congenital bilateral upper limb amelia [1].

Amelia, defined as the congenital absence of one or more limbs, is a rare anomaly with an estimated incidence of 1.5 per 100 000 live births (Fig. 1) [1, 2]. It may occur in isolation or as part of multisystem syndromes and has been linked to both environmental and genetic etiologies. In bilateral upper limb amelia, scoliosis is almost universally observed, yet the medical literature contains little discussion of surgical correction strategies. This lack of guidance poses a challenge for spine surgeons tasked with balancing deformity correction against the preservation of function.

In adolescent idiopathic scoliosis (AIS), the determination of surgical strategy typically prioritizes coronal and sagittal correction, with cosmesis often a driving factor [3, 4]. For individuals with amelia, however, the situation is fundamentally different: upper limb absence necessitates complete reliance on the lower limbs for all daily living activities. Consequently, lumbar mobility is critical to independence, and a long fusion may carry devastating functional consequences. We present a case of scoliosis correction in bilateral upper limb amelia that demonstrates how surgical decision-making must be reframed in this rare and complex context.

## Case report

A 17-year-old male with congenital bilateral upper limb amelia presented with progressive spinal scoliosis. He had adapted impressively to his disability, using his feet to feed, write, groom, and manipulate electronic devices. He lived independently with minimal assistance and was academically successful. However, in the preceding 2 years, he noted worsening truncal asymmetry, postural imbalance, and concern about potential progression of his scoliosis.

On physical examination, he had no upper extremities, a significant left thoracic rib prominence, and truncal shift. Lumbar flexibility was preserved and neurological examination was normal. His gait was steady and unaided. No cutaneous stigmata were present. Psychologically, he displayed resilience but emphasized that his priority was maintaining independence, even at the cost of cosmetic appearance.

**Figure 2:**
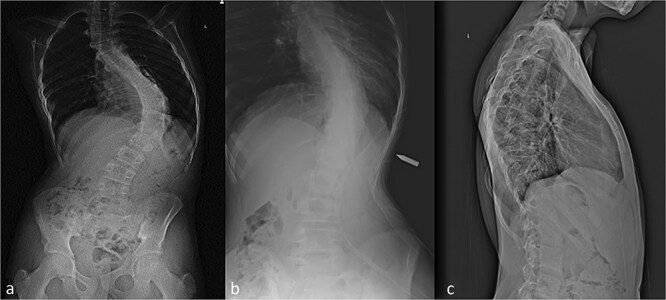
Preoperative whole spine radiographs. AP (a), bolster (b), and lateral (c) views showing 86° thoracic scoliosis with left convexity.

Radiographs demonstrated an 86° left thoracic curve (T6–T12) with skeletal maturity (Risser 5, Sanders 7). The fulcrum unbending Cobb was 66° with only 23% flexibility, confirming a stiff curve. The Harrington stable zone was at L4 and the last touched vertebra was L3, indicating that conventional planning would have required fusion to at least L3. Magnetic resonance imaging excluded intraspinal anomalies (Fig. 2).

Several management options were discussed. Nonoperative management risked continued progression and potential loss of balance. Long posterior fusion from T3 to L3/L4 would likely achieve stable correction but would compromise lumbar mobility essential for his independence. Selective thoracic fusion (STF) offered a compromise, addressing the thoracic curve while sparing the lumbar spine. Anterior scoliosis correction and vertebral body tethering were considered inappropriate given rigidity and skeletal maturity.

The patient and family opted for STF. A posterior approach was used with pedicle screw instrumentation from T3 to L1. Correction was achieved using rod derotation and translation. Four lumbar discs were spared. Neuromonitoring signals remained stable throughout. Postoperative recovery was uneventful, with mobilization on Day 2 and discharge on Day 5.

**Figure 3:**
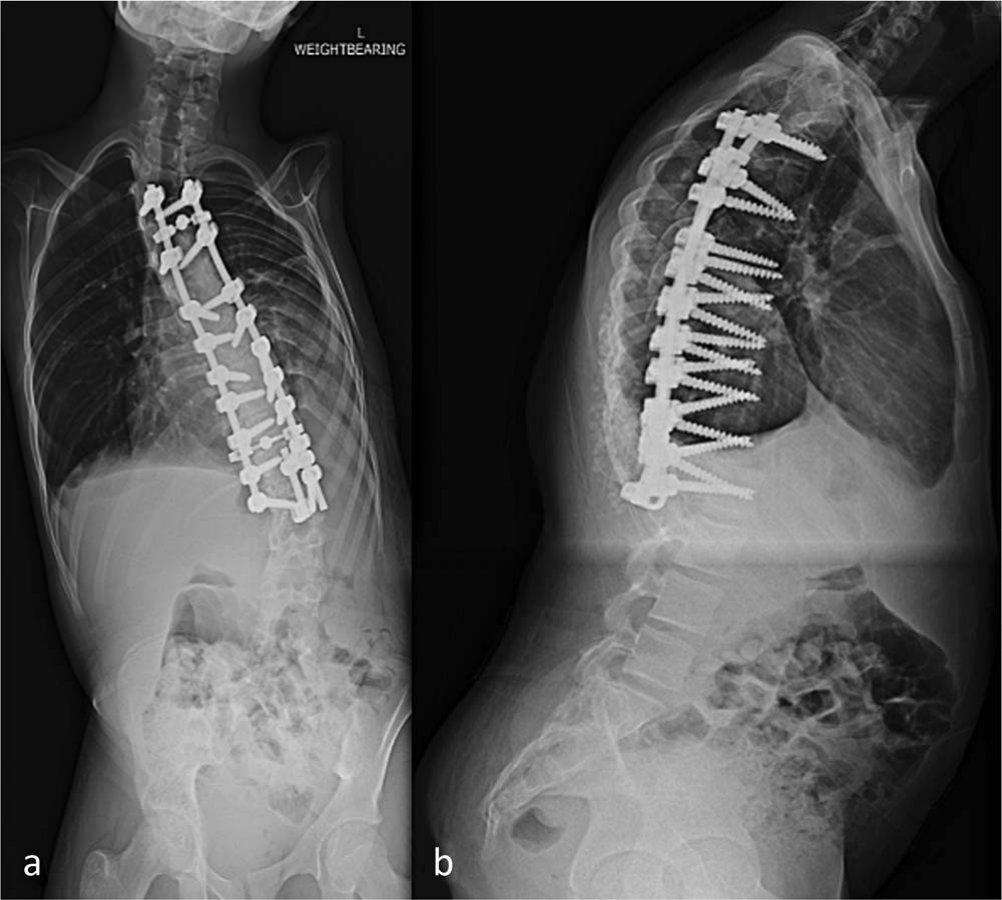
Postoperative whole spine radiographs. AP (a) and lateral (b) views showing improved alignment with Cobb angle reduced to 34° after selective thoracic fusion.

At 2-year follow-up, the Cobb angle improved to 34°. Radiographs demonstrated stable correction without adding-on or coronal decompensation. The patient remained fully independent in all activities of daily living and reported improved posture. His SRS-22 score improved from 2.8 preoperatively to 3.55 postoperatively (Fig. 3).

## Discussion

This case illustrates the rare intersection of scoliosis and bilateral upper limb amelia. In typical AIS management, fusion levels are chosen based on stable vertebra alignment and risk of curve progression. In this patient, theoretical planning suggested fusion to L3 or L4. However, given his reliance on lumbar mobility, such an approach would have undermined his independence.

STF is a recognized strategy in AIS with nonstructural lumbar curves. While the lumbar curve in this case had structural elements, functional considerations outweighed strict radiographic indications. By limiting fusion to T3–L1, we were able to halt progression while preserving mobility critical to independence. Although risks of adding-on or coronal decompensation exist, these did not materialize at 2-year follow-up [5].

Alternative motion-preserving techniques such as vertebral body tethering were unsuitable due to curve stiffness and skeletal maturity. In younger patients with more flexible curves, such options might be considered. For this patient, however, posterior fusion was the only viable intervention [6, 7].

This case also highlights broader ethical considerations. For many adolescents with scoliosis, cosmetic outcome is a dominant concern. Here, the patient prioritized autonomy and independence. The decision-making process underscores the importance of tailoring surgical goals to patient-specific values.

The long-term outlook remains uncertain, with risks of adjacent segment degeneration and coronal imbalance requiring ongoing surveillance. Nonetheless, the positive 2-year outcome suggests that STF can be an appropriate compromise in this rare population. This report contributes to the limited body of literature and underscores the necessity of multidisciplinary planning that integrates surgical, rehabilitative, and psychosocial expertise.

## References

[1] Centers for Disease Control and Prevention 4.9c Limb Deficiency Amelia (Q71.0, Q72.0, Q73.0) . Chapter 4: Diagnosing and Coding Congenital Anomalies. In: Birth Defects Surveillance Toolkit. U.S. Department of Health & Human Services, 2020. https://archive.cdc.gov/www_cdc_gov/ncbddd/birthdefects/surveillancemanual/chapters/chapter-4/chapter4.9c.html (24 September 2025, date last accessed).

[2] Bunnell WP . The natural history of idiopathic scoliosis before skeletal maturity. Spine (Phila Pa 1976) 1986;11:773–6. 10.1097/00007632-198610000-00003 3810290

[3] Winter RB, Moe JH, Wang JF. Congenital scoliosis: a study of 234 patients treated and untreated. J Bone Joint Surg Am 1968;50:1–15. 10.2106/00004623-196850010-00001

[4] Lenke LG, Betz RR, Harms J et al. Adolescent idiopathic scoliosis: a new classification to determine extent of spinal arthrodesis. J Bone Joint Surg Am 2001;83:1169–81. 10.2106/00004623-200108000-00006 11507125

[5] Newton PO, Parent S, Marks M et al. Selective thoracic fusion in adolescent idiopathic scoliosis: a prospective 10-year follow-up. Spine (Phila Pa 1976) 2005;30:S193–203.

[6] Karol LA . The natural history of scoliosis. J Bone Joint Surg Am 2014;96:116–23.

[7] Sponseller PD, Lenke LG, Newton PO et al. When and how much fusion to perform for adolescent idiopathic scoliosis. J Bone Joint Surg Am 2019;101:1502–10.

